# Responses of primary human nasal epithelial cells to EDIII-DENV stimulation: the first step to intranasal dengue vaccination

**DOI:** 10.1186/s12985-016-0598-z

**Published:** 2016-08-18

**Authors:** Nattika Nantachit, Panya Sunintaboon, Sukathida Ubol

**Affiliations:** 1 Department of Microbiology, Faculty of Science, Mahidol University, Bangkok, 10400 Thailand; 2 Department of Chemistry, Faculty of Science, Mahidol University, Bangkok, 10400 Thailand

**Keywords:** Domain III of dengue virus, Trimethyl chitosan nanoparticles, Nasal stimulation, Dengue vaccine

## Abstract

**Background:**

About half of the world’s population are living in the endemic area of dengue viruses implying that a rapid-mass vaccination may be required. In addition, a major target of dengue vaccine are children, thus, a needle-free administration is more attractive. These problems may be overcome by the alternative route of vaccination such as topical, oral and intranasal vaccination. Here, we investigated the possibility to deliver a dengue immunogen intranasally, a painless route of vaccination. The tested immunogen was the domain III of dengue serotype-3 E protein (EDIII-D3) loaded into trimethyl chitosan nanoparticles (EDIII-D3 TMC NPs). The primary human nasal epithelial cells, HNEpCs, were used as an *in vitro* model for nasal responses.

**Results:**

At tested concentrations, EDIII-D3 TMC NPs not only exerted no detectable toxicity toward HNEpC cultures but also efficiently delivered EDIII-D3 immunogens into HNEpCs. Moreover, HNEpCs quickly and strongly produced proinflammatory cytokines (IL-1β, IL-6, TNF-α), type-I IFN, the growth factors (GM-CSF, IL-7), the chemokines (MCP-1, MIP-1β, IL-8), Th1-related cytokines (IL-2, IL-12p70, IL-17, IFN-γ) and Th2-related cytokine (IL-4) in response to EDIII-D3 TMC NPs treatment.

**Conclusions:**

A potential mucosal delivery system for dengue immunogens was revealed and found to stimulate a strong local innate antiviral response which possibly leading to a systemic adaptive immunity.

## Background

Dengue is a viral disease endemic to most areas of Southeast Asia and other subtropical/tropical regions. Infection with any one of the four antigenically related serotypes can cause not only a mild disease, namely dengue fever, but also the more severe illnesses dengue hemorrhagic fever and dengue shock syndrome [1]. The risk of infection is increasing yearly due to the quick and efficient spreading of its vector. Despite tremendous efforts, both prevention and treatment of dengue virus infection are still not effectively addressed because only a vaccine that partially protects adults and adolescents is currently licensed in a few countries. This situation emphasizes the need for an innovative platform for dengue vaccine. Since children are the main targets for dengue vaccination, fear and dislike of injection may be an obstacle for vaccine uptake. Therefore, a needle-free vaccine is a challenge that needs to be addressed.

Nasal immunization, a non-invasive needle-free route, offers various benefits, such as having a highly vascularized and large adsorption surfaces with low proteolysis [2]. It effectively elicits immune response for both systemic and local immunities which may increase the capability of controlling pathogens at the site of entry [3]. The epithelial layer of nasal mucosa is associated with the immunologically active nasal-associated lymphoid tissue (NALT). The nasal lymphoid follicles in the NALT are home to several immunological cells, such as dendritic cells (DCs), T lymphocytes and B lymphocytes, which are all contributing to the induction of the immune response. Interaction between pathogens and the epithelial barrier through pathogen-recognition receptors (PRRs) stimulates epithelial cells to release various cytokines and chemokines, leading to the recruitment of NALT-resident immune cells and subsequent activation of the local and systemic immune responses. In addition, the nasal epithelial layer contains non-ciliated micro-fold cells (M-cells) which are mainly responsible for the particulate uptake and subsequent delivery to the sub-mucosal lymphoid tissues [4]. This will increase the delivery of antigens to lymphoid tissues in the nasal system. Unfortunately, most, if not all, soluble proteins are not well absorbed and are rapidly removed from the nasal cavity by nasal clearance. To circumvent this hurdle, bioadhesive delivery systems have been proposed [5].

Critical issues for effective nasal vaccination are the antigen-retention period that enables its interaction with the lymphatic system and the choice of adjuvant that is nontoxic and induces the required immune responses. Nanoparticles (NPs) of chitosan and trimethyl chitosan (TMC) are considered as one of the most attractive nasal delivery systems as it offers safety, biodegradability, biocompatibility, mucoadhesion, penetration enhancement and adjuvanticity [6, 7]. Therefore, chitosan and TMC NPs have recently been used in the development of vaccines through nasal applications [8–12]. These NPs elicit strong immune response as shown by the significant increase in circulating antibody and the titer of secretory IgA in nasal washes [11, 13, 14].

The envelope (E) protein, the major structural protein on the surface of dengue virus (DENV) particles, is the most promising target for vaccine development. It consists of three domains, namely domain I (EDI) to III (EDIII). Antibodies against EDIII are serotype specific and exert potent neutralizing activity [15]. Herein, EDIII is considered to be relevant in the perspective of creating an efficient vaccine [16, 17]. In conjugation with the adjuvanticity and delivery properties of TMC, EDIII-loaded TMC nanoparticles can plausibly be an effective nasal stimulation vaccine.

In this report, we present a nasal nanoparticle-based dengue immunogen, EDIII-D3 TMC NPs, constructed from the encapsulation of the domain III of dengue serotype-3 E protein (EDIII-D3) into TMC nanospheres. The effect of EDIII-D3 TMC NPs on nasal stimulation was then investigated using HNEpCs as a model. We found that TMC NPs acted as a potent delivery platform for EDIII-D3 which in turn powerfully stimulated the epithelial cell responses. Our study indicates that EDIII-D3 TMC NPs offer an alternative approach of nasal dengue vaccine.

## Methods

### Preparation of secreted E-domain III-dengue virus type 3 protein

The EDIII gene of DENV-3 was cloned into pPICZαB vector and expressed in *Pichia pastoris* as previously described [18, 19]. To obtain secreted EDIII-D3 (sEDIII-D3) protein, the suspension culture of transformed *P. pastoris* was activated by 1 % methanol at 30 °C for 3 days. The culture medium was harvested and concentrated using membrane filtration. The sEDIII-D3 was purified using affinity column chromatography. The purified sEDIII-D3 was confirmed by immunoblotting using EDIII specific antibody and anti-polyhistidine antibody.

### Nanoparticles formulation and characterization

The EDIII-D3 TMC NPs and TMC NPs were formulated using ionic gelation as previously described with minor modifications [20]. To prepare TMC NPs, an aqueous solution of TMC (3.41 mg/ml) containing 0.5 % (w/w) Tween 80 was prepared in HEPES buffer. Subsequently, a solution of 1 mg/ml sodium tripolyphosphate (TPP) was slowly added drop-wise to the TMC solution under constant stirring. EDIII-D3 loaded TMC NPs were prepared by dissolving sEDIII-D3 (0.8 mg/ml) in TPP solution containing 0.5 % (w/w) Tween 80 before mixing with the TMC solution. The formulated TMC NPs and EDIII-D3 TMC NPs were washed three times by being redispersed in HEPES buffer and centrifuged in a Nanosep centrifugal device 100 K (Pall corporation) at 10,000x g for 15 min. The NPs captured in the membrane were redispersed in HEPES buffer. The particle size and zeta-potential were determined using Zetasizer (Nano-ZS, Malvern Instrument, UK).

### Cytotoxicity assay

The primary human nasal epithelial cells, HNEpCs, were purchased from PromoCell, Germany (C-12620). HNEpCs were cultured using commercially available airway epithelial cell growth medium with supplements (C-21060, PromoCell) at 37 °C, 5 % CO_2_. Cells were grown in tissue culture flasks coated with purified collagen (50 μg/ml) (Advanced BioMatrix). The culture medium was refreshed on every other day. The confluent monolayers of HNEpCs were washed twice with PBS before being treated with various concentrations of TMC NPs or EDIII-D3 TMC NPs (25 to 150 μg). HNEpCs cell viability was quantitated using trypan blue exclusion.

### Cellular uptake of EDIII-D3 TMC NPs

HNEpCs cellular uptake of nanoparticles was performed by the previously described method [21]. HNEpCs cultures were treated for 2 days with various concentrations of EDIII-D3 TMC NPs (25 to 112.5 μg) or with sEDIII-D3 (25 μg). At 24 and 48 h of treatment, cells were washed, fixed and permeabilized using Cytofix/Cytoperm (BD Biosciences). The intracellular EDIII-D3 was stained with anti-EDIII specific antibody. The uptake was evaluated by measuring the mean fluorescence intensity (MFI) of cells and the percentage of fluorescence positive cells.

### Cytokines and chemokines production

HNEpCs cultures were washed with PBS before being treated with TMC NPs, EDIII-D3 TMC NPs or sEDIII-D3 for 48 h. Aliquots of supernatant were harvested at 24 and 48 h. Harvested supernates were subjected to cytokine and chemokine quantification using Bio-Plex bead based assay (Bio-Rad Laboratories), following the manufacturer’s instruction. Seventeen cytokines and chemokines (IL-1β, IL-6, TNF-α, G-CSF, GM-CSF, IL-7, MCP-1, MIP-1β, IL-8, IL-2, IL-12p70, IL-17, IFN-γ, IL-4, IL-5, IL-10, IL-13) were quantitated simultaneously. An antiviral cytokine, IFN-α, was measured separately using a commercially available kit (VeriKine™ human IFN-alpha, PBL interferon source).

### Statistical analysis

All data shown were calculated from at least three independent experiments. Results are expressed as mean ± SD and were analyzed using Statview software. Statistical comparison of cytokine productions among control and test groups were performed using the non-parametric Mann–Whitney *U* test. Results were considered statistically significant at *P* <0.05.

## Results

### Formulation of EDIII-D3 TMC nanoparticles

As shown in Table 1, the average size of the established empty TMC NPs was 225.9 ± 4.5 nm and they were positively charged (+33.4 ± 0.1 mV). When sEDIII-D3 was encapsulated into TMC NPs, a slight enlargement of the particles and a reduction of zeta-potential were observed (255.1 ± 4.2 nm and +25.5 ± 0.4 mV). This may be attributed to the interaction between positively charged TMC and the negatively charged sEDIII-D3.

**Table 1:** Characteristics of nanoparticles

Nanoparticles	Particle size (nm)	Polydispersity index	Zeta-potential (mV)
EDIII-D3 TMC NPs	255.1 ± 4.2	0.211 ± 0.016	+25.5 ± 0.4
TMC NPs	225.9 ± 4.5	0.133 ± 0.013	+33.4 ± 0.1

Formulation of EDIII-D3 TMC NPs and TMC NPs was based on ionic gelation. Their properties, including size, polydispersity index and zeta-potential, were measured by Zetasizer. Results from three independent experiments were expressed as mean ± SD

### Responses of primary human nasal epithelial cells to EDIII-D3 TMC NPs treatment

**Fig. 1:**
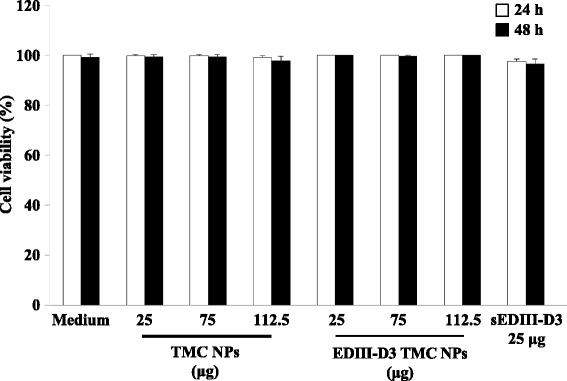
Viability of HNEpCs treated with TMC NPs or EDIII-D3 TMC NPs. HNEpCs were treated with TMC NPs or EDIII-D3 TMC NPs at concentrations of 25 to 150 μg for 2 days. Cell viability was monitored using the trypan blue exclusion assay. The results obtained were compared with that of the untreated cultures. All values are represented as mean ± SD

The HNEpCs cultures were treated with TMC NPs, or EDIII-D3 TMC NPs or sEDIII-D3 for two days. The cytotoxicity, nanoparticle internalization and antiviral responses were assessed at 24 and 48 h of treatment. Cell viabilities were 99.4 ± 0.9 % and 94.9 ± 2.8 %, for 24 and 48 h of treatment, respectively (Fig. 1). This result indicated that, at the tested concentrations, nanoparticles showed no toxicity toward HNEpCs.

**Fig. 2:**
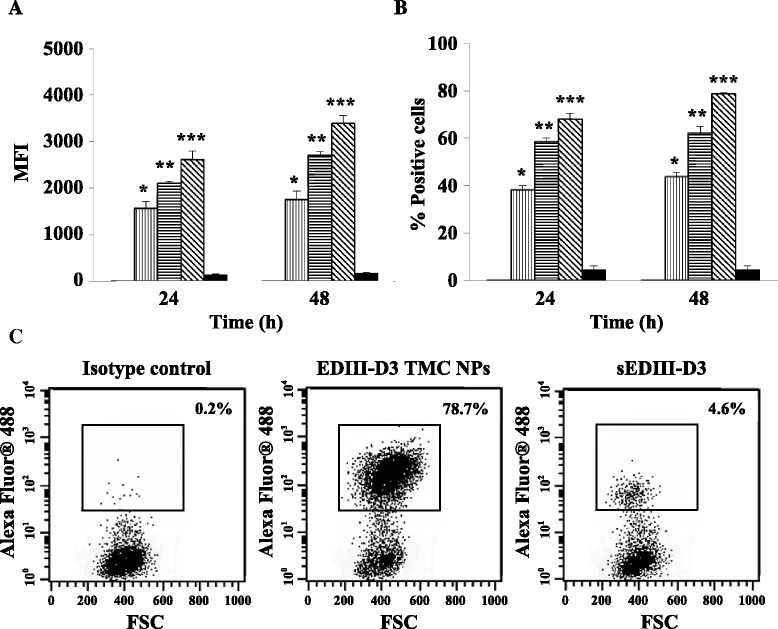
Delivery of EDIII-D3 into HNEpCs. HNEpCs cultures were treated with various amounts of EDIII-D3 TMC NPs or 25 μg of sEDIII-D3 for 2 days prior to the intracellular staining with a monoclonal anti-EDIII specific antibody. The mean fluorescence intensity (MFI) (**a**) and the percentage of fluorescence-positive cells (**b**) were quantitated by flow cytometry. Dot plot analysis of HNEpCs treated with isotype control, EDIII-D3 TMC NPs at 112.5 μg and sEDIII-D3 at 25 μg were shown from one representative out of three independent experiments (**c**). *, ** and *** indicate significant differences between 25, 75 and 112.5 μg of EDIII-D3 TMC NPs and 25 μg of sEDIII-D3 (*P* <0.05).  Medium,  EDIII-D3 TMC NPs (25 μg),  EDIII-D3 TMC NPs (75 μg),  EDIII-D3 TMC NPs (112.5 μg) and  sEDIII-D3 (25 μg)

We then investigated the efficiency of nanoparticle uptake by HNEpCs. As revealed in Fig. 2a-c, the cultures treated with the highest dose of EDIII-D3 TMC NPs (112.5 μg) showed the strongest fluorescence intensity (MFI) and had the highest percentage of positive cells. In contrast, treatment with 25 μg of sEDIII-D3 demonstrated the lowest MFI and percentage of positive cells. These results indicated that TMC NPs effectively delivered EDIII-D3 to HNEpCs.

**Fig. 3:**
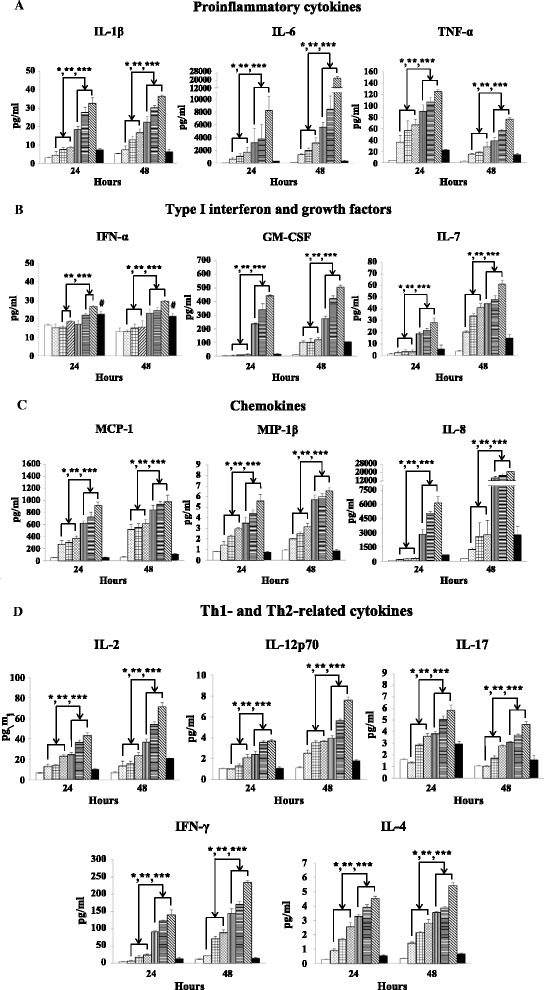
EDIII-D3 TMC NPs treatment induces the expression of cytokines and chemokines by HNEpCs. Cultures of HNEpCs were treated with TMC NPs (25, 75, 112.5 μg) or EDIII-D3 TMC NPs (25, 75, 112.5 μg) or 25 μg of sEDIII-D3. Aliquots of supernatant were harvested at 24 and 48 h of treatment. Cytokines and chemokines were quantitated using Bio-Plex bead-based assay as well as IFN-α ELISA assay. The mean production of proinflammatory cytokines (**a**), antiviral cytokine and growth factors (**b**), chemokines (**c**), Th1- and Th2-related cytokines (**d**) are presented. All values are expressed in pg/ml. The experiment was performed in three independent replicates. *, ** and *** indicate significant differences between the amounts of cytokines measured for 25, 75 and 112.5 μg of EDIII-D3 TMC NPs and TMC NPs (*P* <0.05). ^**#**^ indicates significant differences between 25 μg of sEDIII-D3 and mock-treated cultures (*P* <0.05).  Medium,  TMC NPs (25 μg),  TMC NPs (75 μg),  TMC NPs (112.5 μg),  EDIII-D3 TMC NPs (25 μg),  EDIII-D3 TMC NPs (75 μg),  EDIII-D3 TMC NPs (112.5 μg) and  sEDIII-D3 (25 μg)

The immune stimulatory effect of EDIII-D3 TMC NPs was subsequently investigated. In EDIII-D3 TMC NPs treated HNEpCs, the production of the proinflammatory cytokines (IL-1β, IL-6 and TNF-α) was stronger than that of cells treated with other systems (Fig. 3a). The highest concentrations of IL-1β (36.5 ± 0.8 pg/ml) and IL-6 (22,949.1 ± 2,154.9 pg/ml) were observed at 48 h. Interestingly, EDIII-D3 TMC NPs transiently activated TNF-α production, as evidenced by the sharp increase in TNF-α at 24 h (125.4 ± 2.9 pg/ml), before it decreased to the baseline level by 48 h. In contrast, sEDIII-D3 did not stimulate IL-1β, IL-6 and TNF-α production. This result indicated that the immunogenicity of sEDIII-D3 was greatly enhanced by TMC NPs.

Of note, the production of the antiviral type-I interferon was significantly increased once HNEpCs were treated with EDIII-D3 TMC NPs or sEDIII-D3 but not with empty TMC NPs (Fig. 3b). This suggested that the activation of type-I IFN production by EDIII-D3 TMC NPs resulted from the direct antigenic effect of the encapsulated EDIII-D3. A pronounced stimulatory effect on growth factor (GM-CSF and IL-7) production was observed after incubation of HNEpCs with EDIII-D3 TMC NPs when compared to that of other treatments (Fig. 3b). As shown in Fig. 3b, the EDIII-D3 TMC NPs significantly stimulated the production of GM-CSF and IL-7 in a dose- and time-dependent fashion.

The ability to induce chemokine production was shown in Fig. 3c. An obviously upregulated production of MCP-1, MIP-1β and IL-8 was detected since 24 h of treatment (Fig. 3c). This response was continuously increased up to 48 h of treatment. Remarkably, the EDIII-D3 TMC NPs is a potent inducer of IL-8 (21,072.9 ± 174.2 pg/ml).

For the Th1-related cytokines, EDIII-D3 TMC NPs markedly stimulated the secretion of IL-2, IL-12p70, IL-17 and IFN-γ when compared to that of other conditions (Fig. 3d). The stimulatory effect of EDIII-D3 TMC NPs on IL-2, IL-12p70, IL-17 and IFN-γ production was observed from 24 h onwards. The production of Th2-related cytokine, IL-4, was upregulated since 24 h of treatment (Fig. 3d) while IL-10 was undetectable (data not shown).

## Discussion

An alternative route of vaccination, such as intranasal, oral, topical, vaginal and rectal route, is gaining attention for the vaccine market. Among these routes, the nasal route offers the most promising opportunity for vaccine administration. In regard of dengue vaccine, about half of the world’s population is living in the endemic areas of DENV suggesting that a global vaccination may be required. It is clear that intranasal vaccination facilitates greater public compliance and a rapid mass vaccination. With these reasons, it may be worth to develop a needle-free intranasal platform of dengue vaccine.

Intranasal vaccination can induce a broad immune response leading to the production of IgG, mucosal IgA antibodies and to the activation of cytotoxic T-lymphocytes. Therefore, it may protect against not only the mucosal viruses [22–28] but also the systemic infection. The feasibility for the delivery of dengue immunogen through the mucosal route was performed using the live EDIII-producing *Lactococcus lactis* as the delivery system [29]. Sim A.C., et al. found that both oral and intranasal administrations triggered a systemic anti-DENV neutralizing antibody [29]. This indicated that mucosal administration of dengue immunogens is able to activate systemic immune responses suggesting the potential use of the needle-free vaccination against DENV.

We describe here the use of HNEpCs as an in vitro model for testing an intranasal delivery of dengue immunogen. We found that HNEpCs are permissive to EDIII-D3 delivery *via* TMC NPs. The effective internalization of EDIII-D3 may be mediated through the interactions between the positively charged EDIII-D3 TMC NPs and negatively charged cell membranes [30].

To control virus infection, both innate and adaptive immune responses have to work in harmony. In our present study, the capability of EDIII-D3 TMC NPs to trigger innate and adaptive immune responses was demonstrated through the production of proinflammatory cytokines (IL-1β, IL-6, TNF-α), the antiviral cytokine (IFN-α), growth factors (GM-CSF, IL-7), Th1-related cytokines (IL-2, IL-12p70, IL-17, IFN-γ), Th2-related cytokine (IL-4) and chemokines (MCP-1, MIP-1β, IL-8) by HNEpCs. We revealed that EDIII-D3 TMC NPs were strong inducer of these mediators. The significant increase in IL-1β production indicates that EDIII-D3 TMC NPs are able to activate inflammasome. Recent publications have highlighted the importance of inflammasome activation in the control of viruses including chikungunya virus and HIV [31, 32]. Moreover, the antiviral activity can be amplified by the chemoattractant function of the chemokines which recruit leukocytes to the site of infection and activate these cells to secrete the proinflammatory cytokines (TNF-α, IL-1β and IL-6). This result suggested that EDIII-D3 TMC NPs initiated an acute phase anti-viral response.

An upregulation of type I interferon production upon EDIII-D3 TMC NPs stimulation is of interest. In general, type I interferon gene is stimulated through the interactions between the DNA or RNA of the invaders and the pattern recognition receptors [33]. In our tested system, EDIII protein was an immunogen. How EDIII protein activates type I interferon production remains unclear. However, NS1 protein of DENV is reported to be able to induce type I interferon production upon its interaction with TLR-4 [34]. Whether EDIII protein uses a similar pathway of activation requires further investigation.

Besides the innate response, adaptive immune responses may be stimulated upon EDIII-D3 TMC NPs treatment. This was shown by the upregulation of Th1- and Th2-related cytokine production (IL-2, IL-12p70, IL-17, IFN-γ and IL-4). These two groups of cytokine activate T cell proliferation as well as B cell differentiation into plasma cells which are the major effectors of viral clearance.

In summary, the observed expression profiles of cytokines and chemokines demonstrate the ability, at least in an in vitro model, of the EDIII-D3 TMC NPs to initiate the cellular processes essential for the induction of immunity including the activation of monocytes, macrophages and neutrophils, the triggering of acute phase responses, the formation of inflammasome complex, the enhanced expansion of naïve and memory CD4 T cells, and the growth proliferation and differentiation of T cells.

The response of human monocyte-derived dendritic cells (MoDCs) to the same delivery platform (EDIII-D3 TMC NPs) was also investigated (Nantachit et al., manuscript in preparation). We found that MoDCs effectively internalize and strongly respond to EDIII-D3 TMC NPs. Therefore, it would be interesting to investigate whether intranasal administration of EDIII-D3 TMC NPs would stimulate immunological cells in the nasal-associated lymphoid tissue. The mediators produced from the activated nasal epithelium may directly stimulate neighboring DCs and NALT-resident immunocytes [35]. In addition, the mucoadhesive property of TMC NPs may prolong the residence time of the antigen at the nasal mucosa and allow particles to be uptaken by M-cells and transcytosed to the posterior lymph nodes, where they can initiate immunity [36].

## Conclusions

We have demonstrated the feasibility of using EDIII-D3 TMC NPs as a vehicle for the delivery of a dengue immunogen in nasal epithelial cells. Delivery of the immunogen *via* this route strongly upregulated the production of antiviral cytokine, inflammatory cytokines, Th1- and Th2-related cytokines by nasal epithelial cells. This platform of dengue vaccine can be further developed by co-encapsulation of immunopotentiator with the multiple types of dengue antigen into TMC NPs if more immunogenicity would be required. Thus, it is deserved to be further investigated as an alternative painless dengue vaccine for children.

## Abbreviations

DCs: Dendritic Cells
DENV: Dengue Virus
E: Envelope
EDIII-D3 TMC NPs: The Domain III Of Dengue Serotype-3 Envelope Protein Loaded into Trimethyl Chitosan Nanoparticles
EDIII-D3: The Domain III Of Dengue Serotype-3
HNEpCs: Human Nasal Epithelial Cells
M-cells: Micro-Fold Cells
MFI: Mean Fluorescence Intensity
MoDCs: Monocyte-Derived Dendritic Cells
NALT: Nasal-Associated Lymphoid Tissue
NPs: Nanoparticles
PRRs: Pathogen-Recognition Receptors
sEDIII-D3: Secreted Domain III of Dengue Serotype-3
TMC: Trimethyl Chitosan
TPP: Sodium Tripolyphosphate
